# Stop it! Relationship between sport expertise and response inhibition in elite athletes

**DOI:** 10.3389/fpsyg.2023.1192483

**Published:** 2023-06-05

**Authors:** Marie-Therese Fleddermann, Lukas Reichert, Björn Wieland, Karen Zentgraf

**Affiliations:** Department of Movement Science and Training in Sports, Institute of Sport Sciences, Goethe University Frankfurt, Frankfurt am Main, Germany

**Keywords:** SSRT, SST, executive functions, sport games, open-skill sport

## Abstract

**Introduction:**

The dynamic structure of sport games forces players to make time-sensitive decisions and to initiate actions that may then have to be canceled in response to sudden changes in the game situation. Whether and up to which time already initiated movements can still be inhibited is an important criterion for game performance in elite sport. Research indicates that elite athletes show superior motor inhibition performance compared to recreational athletes. However, no study has examined whether differences also emerge among professional elite athletes themselves. Therefore, this study aimed to investigate whether motor inhibition performance is a differential feature among elite athletes, and whether inhibition performance increases with greater expertise.

**Methods:**

In total of 106 elite athletes (ice hockey, basketball, volleyball, American football, handball, and soccer) completed a PC-based procedure to determine motor inhibition performance using the stop-signal reaction time (SSRT) task for hands and feet. In addition, an expertise score was determined for each elite athlete. Multiple linear regression was used to calculate the relationship between expertise and SSRT.

**Results:**

Results showed that the expertise score of the elite athletes was between 3.7 and 11.7 out of 16 possible points (*M*_Expertise_ = 6.8 points, *SD* = 1.76). The average SSRT of the hands was 224.0 ms (*SD* = 35.0); of the feet, 257.9 ms (*SD* = 48.5). Regression results showed a significant relationship between expertise and SSRT (*F*_(2,101)_ = 9.38, *p* = 0.04, *R*^2^ = 0.06). SSRTs of the hands were significant predictors of expertise (*b* = −0.23, *t* = −2.1, *p* = 0.04).

**Discussion:**

Taken together, results suggest that elite athletes with higher expertise outperform elite athletes with lower expertise, indicating that it is possible to differentiate within elite athletes with respect to inhibition performance of the hands. However, whether expertise affects inhibition performance or vice versa cannot be answered at present.

## 1. Introduction

Inhibitory control, which is described as a key component of executive functions, seems to be highly relevant for sports performance (Verburgh et al., 2014; Vestberg et al., 2017, 2020). It can be divided into three categories: interference control (responding to distracting stimuli), impulse control (giving no response), and response inhibition (inhibiting an already initiated response) (Albaladejo-Garcia et al., 2023). Especially the last category, “response inhibition”, seems to be important for success and performance in different sports (Vestberg et al., 2012; Verburgh et al., 2014). This is particularly the case in so-called open-skill sports (Zhu et al., 2020) in which athletes perform in permanently changing and challenging situations in which they have to make quick decisions coupled with fast actions, while sometimes suddenly having to interrupt their action due to a new playing situation. For example, in a basketball offense situation, the point guard decides, based on the current playing situation, to play a pass to an open teammate. But between the decision and the motor execution of the pass, the game situation changes suddenly, and the teammate is no longer open (e.g., an opponent player has moved into the passing lane). In this case, the point guard must inhibit the already initiated movement to avoid a turnover. Hence, not only the general ability of response inhibition, but also up to which time an already initiated movement can be stopped seem to be important.

Especially in elite sports, being able to inhibit a movement at a late time seems beneficial, because of very dynamic and fast-changing playing situations as well as time-critical movements. In this vein, some studies have examined whether elite athletes perform better on cognitive tasks (Mann et al., 2007; Voss et al., 2010; Heppe et al., 2016) and in a response-inhibition task compared to non-athletes (Zhang et al., 2015; Brevers et al., 2018; Bravi et al., 2022) and recreational athletes. Most studies showed that elite athletes perform better in a response inhibition task than recreational athletes. For example, Heppe and Zentgraf (2019) examined elite handball players (2nd German league) and sport students. They found shorter so-called stop-signal reaction times (SSRTs, Verbruggen and Logan, 2008) in elite players, which means that elite athletes needed less time to inhibit an already initiated movement than active controls (physical education students). Similar results have been reported in other types of sport such as soccer (Verburgh et al., 2014; Huijgen et al., 2015) or in different sport categories such as golf, rugby, basketball, and soccer (Hagyard et al., 2021). In contrast, however, some studies in the field also reported no differences between either elite athletes and recreational athletes (Wang et al., 2013) or athletes and non-athletes (Chan et al., 2011). One possible explanation for these inconsistencies could be the structure of the sport task itself. This could mean that only athletes acting permanently in complex and dynamic environments benefit from their sport practice and develop greater inhibition performance compared to athletes acting in less complex environments. This explanation is supported by Wang et al. (2013) study of inhibition performance in athletes from different sport categories. Their findings suggest that athletes acting in open-skill sports such as tennis show shorter SSRTs than athletes acting in close-skill sports such as swimming. This could indicate that sport practice in challenging and dynamic environments relates to performance in response inhibition. This is supported by Zhu et al. (2020) meta-analysis of the effects of open-skill exercise on inhibition performance. They found greater benefits from open-skill exercise than closed-skill exercise. They argued that open-skill exercise mobilizes more cognitive resources, possibly accompanied by functional and structural changes in the brain resulting in improved executive function performance. Moreover, studies in other cognitive domains (e.g., on processing speed, anticipation, visual search) show benefits for open-skill sports and support the notion that highly demanding environments in complex sports (with opponents, unpredictable situations, fast actions, etc.) facilitate the development of cognitive functions (for overviews, see Mann et al., 2007; Voss et al., 2010; Gu et al., 2019; Heilmann et al., 2022).

Drawing on these conclusions on the practice and development of inhibitory control, Heppe and Zentgraf (2019) additionally investigated whether effects may be specific to the motor effectors. They examined inhibition performance not only with hands but also with feet in both elite handball players and physical education students. Results of this motor-effector specificity showed that elite handball athletes performed much better with their hands compared to recreational athletes, but no significant differences between groups were detected when executing the task with their feet. The authors suggested that handball players may not be better *per se* in response inhibition, but may benefit from handball practice, which is characterized by techniques performed mostly with their hands (catching, throwing, dribbling).

In sum, the type of sport (open-skill vs. closed-skill exercise) seems to be relevant and may enhance motor inhibition performance. Nonetheless, time and level of sport practice could also exert a positive influence. This means that with increasing expertise level, the requirements in sport practice and the effects of sport practice will also increase. This is shown for example in some studies in volleyball (Alves et al., 2013; Formenti et al., 2022), showing greater cognitive functions in athletes with higher expertise. An open question is whether response inhibition performance differentiates not only between elite and recreational athletes but also between individual elite athletes—for example, the higher the expertise level of an athlete, the better the inhibition performance. A general issue here, however, is the definition of elite athletes. Overall, there is high variance in the classification of expertise with studies using different criteria such as the “ten-year rule” (Ericsson et al., 1993), training hours such as more than 10,000 h (Gladwell, 2008), playing in professional leagues (Ivarsson et al., 2013), or simply the fact of participating in competitions (Voss et al., 2010). However, these definitions are not sufficient to determine differences between experts, because findings are not really comparable across studies and clear criteria on how to define valid samples of experts are lacking. Swann et al. (2015) have suggested defining the elite level on the basis of two categories: (1) within-sport competition and (2) between-sport competition. The first category (eliteness, within-sport competition) includes the athlete's highest standard of performance (A), success on the athlete's highest level (B), and experience on the athlete's highest level (C). The second category (expertise of athletic sample, between-sport competition) consists of the competitiveness of the sport in the athlete's country (D) and the global competitiveness of the sport (E). In the response inhibition literature, only Hagyard et al. (2021) have used Swann et al. (2015) categorization to describe athletes in more detail. In two studies, they found that superelite athletes (highest score = 13–16 points, based on the formula) outperformed elite athletes (score of 9–12), amateur athletes (score of 5–8 points), and novices (score of 1–4 points) in terms of superior response inhibition performance.

Therefore, the aim of the present study was to examine the relationship between expertise and response inhibition performance in order to gain a clearer picture of expertise effects (especially between elite athletes on the highest level). To apply a differentiated definition of elite athletes, an expertise score was calculated for each athlete based on the recommendations of Swann et al. (2015). Drawing on findings from previous studies (e.g., Hagyard et al., 2021) showing better response inhibition performance in superelite athletes, we expected to find a relationship between expertise score and response inhibition performance. We hypothesized that elite athletes with higher expertise scores would perform better on an inhibition task than athletes with lower expertise scores. Additionally, based on findings of Heppe and Zentgraf (2019), we addressed effector specificity by examining both hands and feet. Depending on the sports type (e.g., when the sport is more hand-dominated), we expected that response inhibition performance in hands is a better predictor of the expertise level compared to feet.

## 2. Material and methods

### 2.1. Participants

One hundred and six elite athletes (*M*_age_ = 24.49 ± 4.83 years, *n* = 45 were woman) from different types of open-skill sports participated in the study (Table 1). These were 19 ice hockey players (*n* = 19 woman), 34 volleyball players (*n* = 18 woman), 38 basketball players (5 vs. 5 and 3 × 3) (*n* = 7 woman), one soccer player (*n* = 1 woman), two American football players, and 12 handball players. All belonged to either the German national team or the German junior national team or they played in one of the two highest German leagues (1st Bundesliga, 2nd Bundesliga). The methods and study protocol were applied according to the Declaration of Helsinki and were approved by the local ethics committee. All participants and, if necessary, their legal guardians (athletes under 18 years) gave written informed consent prior to any data collection.

**Table 1:** Type of sport, number of women, age range, and performance level of all athletes.

**Type of sport**	**Woman**	**Age range between**	**Performance level (national team, junior national team or others)**
All athletes (*n =* 106)	*n =* 45	17–38 years	*n =* 35 National team
			*n =* 33 Junior national team
			*n =* 38 Others (1st, 2nd league)
Volleyball (*n =* 34)	*n =* 18	17–30 years	*n =* 6 National team
			*n =* 14 Junior national team
			*n =* 14 Others (1st, 2nd league)
Ice hockey (*n =* 19)	*n =* 19	20–35 years	*n =* 19 National team
Handball (*n =* 12)	*n =* 0	19–34 years	*n =* 1 National team
			*n =* 4 Junior national team
			*n =* 7 Others (1st, 2nd league)
Basketball (*n =* 38)	*n =* 7	17–38 years	*n =* 9 National team
			*n =* 14 Junior national team
			*n =* 15 Others (1st, 2nd league)
Other sports (Soccer, American football) (*n =* 3)	*n =* 1	23–25 years	*n =* 1 Junior national Team
			*n =* 2 Others (1st, 2nd league)

### 2.2. Procedure and experimental setup

Upon arriving and after being informed about the testing protocol, participants completed a questionnaire about their sports career in order to calculate the expertise score. Afterwards, they completed the response inhibition test with hands and feet. The test session took place in a quiet room. Participants sat on a chair in front of a screen, wore headphones, and always started with detailed instructions, a practice trial, and the test trials in both hand and feet conditions. Half of participants started the test with their hands; the other half, with their feet.

### 2.3. Expertise score

To examine the elite level of the athletes, a questionnaire was created based on Swann et al. (2015) recommendations to use the five variables representing two categories as reported above in the introduction. Each variable was divided into four categories that should differentiate between athletes. All participants completed this questionnaire.

Based on these five variables, an expertise score was calculated using the following formula: ([A+B+C/2]/3) × ([D + E]/2). In addition, Swann et al. (2015) proposed a classification system (for “eliteness”) based on the resulting score: 1–4: semi-athletes; 4–8: competitive athletes; 8–12: successful athletes; and above 12 points: world-class athletes. However, for our analysis, we used only the expertise score and not the four “eliteness” categories.

### 2.4. Response inhibition performance

Response inhibition was assessed with the stop-signal test (SST) paradigm created by Verbruggen and Logan (2008). The paradigm was carried out using the software MATLAB^®^ (version R2020a MATLAB 9.8). The test consisted of two types of stimuli: a “Go” stimulus and a “Stop” stimulus. The “Go” stimulus was a white arrow pointing either to the left or the right side. The instruction for participants was to react as quickly as possible with their hands/feet as soon as they saw the arrow. If the arrow pointed to the left, participants had to press the left button (with the left hand/foot); if to the right, the right button (with the right hand/foot).

The “Stop” stimulus also consisted of a white arrow pointing to the left or the right side, but the white arrow was replaced after a variable delay (stop-signal delay, SSD) by a blue arrow. The instruction to participants was to inhibit their response by not pressing any button if the arrow turned blue. In order to define an individual response inhibition performance, the stop condition adapted automatically via a staircase procedure. If a participant failed in a stop condition (and pushed the button), the next trial was easier due to a shorter SSD (the arrow turned blue 50 ms earlier). On the other hand, if a participant was successful in the stop condition (not pushing the button), the next trial was harder due to a longer SSD (the arrow turned blue 50 ms later). This procedure guaranteed that each participant was tested at her or his own response inhibition performance threshold. The order of the “Go” stimuli (75% of all trials) and “Stop” stimuli (25% of all trials) was randomized for each participant and condition (hands/feet).

Based on the reaction times and the successful stop trials in the stop-signal test, an individual stop-signal reaction time (SSRT) could be calculated for each participant. This has been described as the key measure of inhibition efficiency (Verbruggen and Logan, 2008; Verbruggen et al., 2013; Matzke et al., 2018) and was used as the dependent variable. Based on recommendations of Verbruggen et al. (2013, 2019), we used the integration method to calculate the SSRT. This method uses the point at which the integral of the reaction time distribution is equal to the probability of reaction time (*n*th RT), and calculates SSRT by subtracting the mean SSD from the reaction time (*n*th RT). This method seems to be a reliable method for measuring stop-signal reaction times (Verbruggen et al., 2013, 2019).

### 2.5. Data and statistical analysis

Response inhibition data for hands and feet were analyzed with Verbruggen et al. (2008) analysis script, and expertise scores were calculated for each subject with Microsoft Excel Version 16.10 based on Swann et al. (2015) formula ([A+B+C/2]/3) × ([D + E]/2). Statistical analyses were performed with IBM SPSS Version 29. Means (*M*) and standard deviations (*SD*s) were used to describe participants' expertise score and SSRTs. Pearson's correlation coefficient between SSRT and hands and feet performance was used to examine the relationship between expertise score and SSRTs. Additionally, we calculated the 95% confidence interval [CI]. Finally, a multiple linear regression analysis was conducted to examine the effects of SSRT performance of hands and feet on expertise scores. The distribution of residuals was checked both visually and with a Shapiro-Wilk test. The effect size of the regression analysis is reported with Cohen's d.

## 3. Results

Two elite athletes were excluded because they showed an atypical performance in the SST paradigm that failed to meet the criteria for calculating SSRTs (e.g., reaction times for the hands exceeded 800 ms).

### 3.1. Expertise score

**Figure 1:**
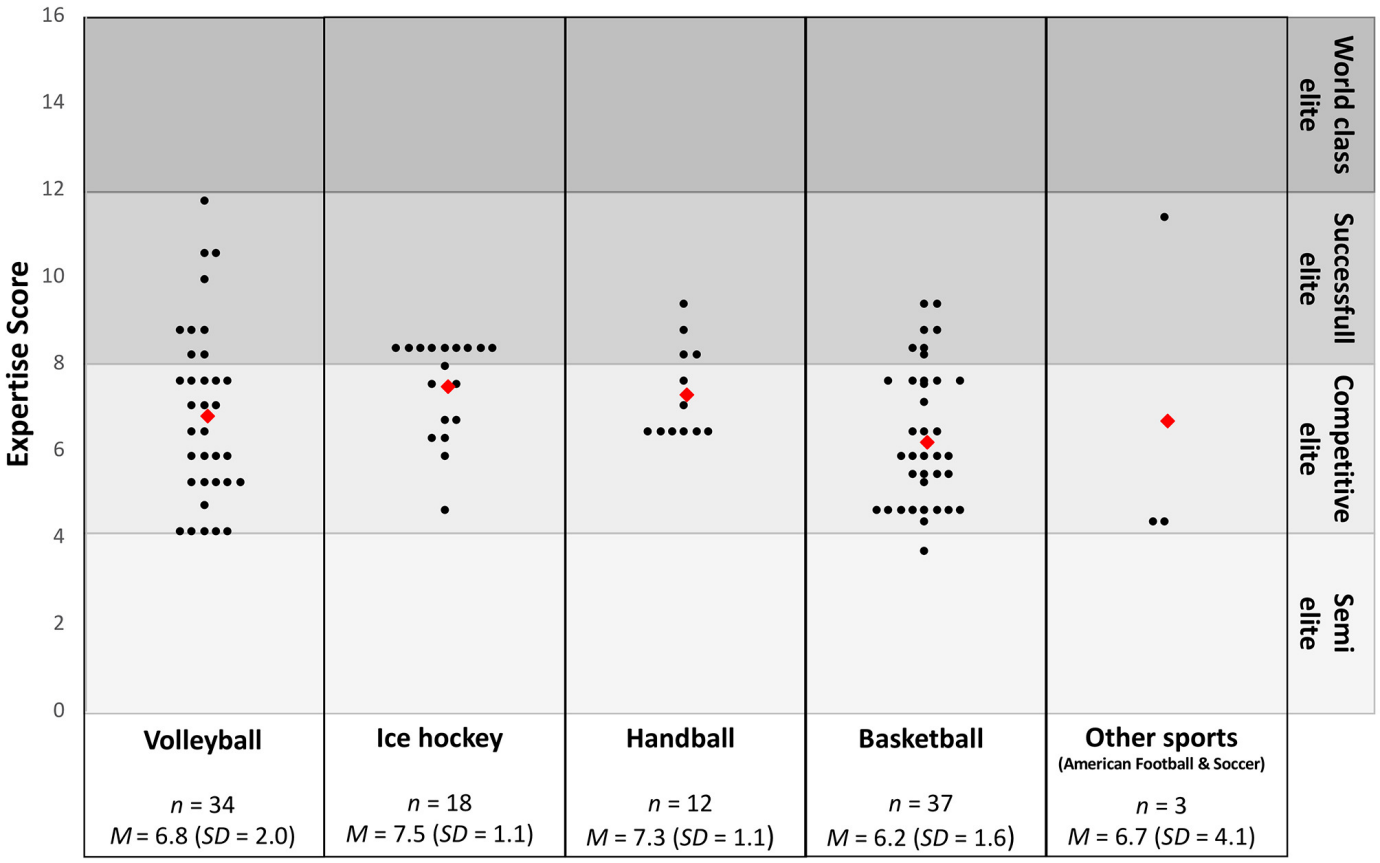
Expertise score in points for each participant (black circle) divided into type of sport (basketball, volleyball, ice hockey, handball) and other sports (American football and soccer) plus *M*_Expertisescore_ (red square) of each type of sport. M, Mean; SD, Standard deviation.

The mean expertise score of all athletes was 6.80 points (*SD* = 1.76) out of a possible 16 points. The minimum score was 3.67 points, and the maximum score was 11.7 points. According to Swann et al. (2015) classification system, one (<1%) athlete could be classified as semi-elite (1–4 points); 72 (69.2%), as competitive elite (4–8 points); and 31 (29.8%), as successful elite (8–12 points). No athlete in our sample could be classified as world-class elite (above 12 points). Figure 1 presents the type of sport, the expertise score, and the distribution across the four categories.

### 3.2. Response inhibition performance and expertise score

Mean response inhibition performance (SSRT) for hands was 224.0 ms (*SD* = 35.0 ms); for feet, 257.9 ms (*SD* = 48.5 ms). Mean reaction time for hands was 501.8 ms (*SD* = 103.3 ms); for feet, 512.0 ms (*SD* = 119.9 ms).

**Figure 2:**
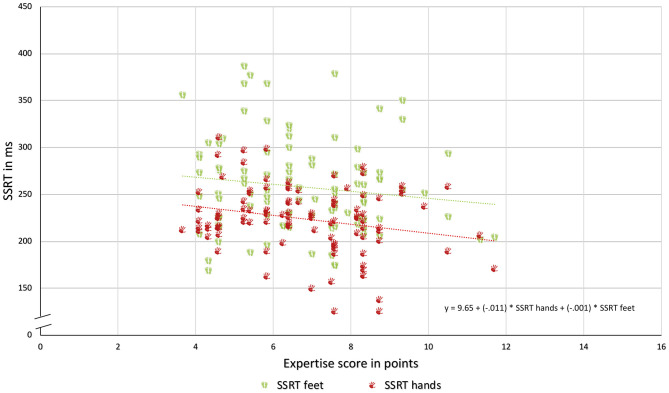
Relationship of expertise score (in points) and SSRTs (in ms) to hands (presented as a red hand) and feet (presented as green feet).

Stop-signal reaction times for hands correlated with the expertise score, Pearson's *r*_(104)_ = −0.24, 95% CI [−0.41, −0.05], *p* = 0.01. Stop-signal reaction times for feet and the expertise score were not correlated, Pearson's *r*_(104)_ = −0.14, 95% CI [−0.32, 0.58], *p* = 0.17. Results are shown in Figure 2.

Results of the multiple linear regression analysis showed a significant relationship between expertise and SSRT [*F*_(2,101)_ = 9.38, *p* = 0.04, *R*^2^ = 0.06, *d* = 0.20) with a significant influence of the hands (*b* = −0.23, *t* = −2.1, *p* = 0.04).

## 4. Discussion

The aim of the present study was to examine the relationship between expertise level and response inhibition performance. We hypothesized that elite athletes with higher expertise would need less time to suppress their response than elite athletes with lower expertise. As predicted, we found a significant relationship between expertise and response inhibition performance indicating shorter SSRTs for hands in high-elite athletes. Additionally, the multiple regression analysis revealed a significant influence for the hands condition. These results are in line with previous studies showing better performance in (elite) athletes compared to recreational athletes (Wang et al., 2013; Verburgh et al., 2014; Huijgen et al., 2015; Heppe and Zentgraf, 2019). Albaladejo-Garcia et al. (2023) also examined expertise and experience effects of different levels of expertise (elite vs. recreational vs. non-athletes) in response inhibition performance in a meta-analysis. They found a small effect size (SMD = 0.44, 95% CI [0.14, 0.73]) with athletes performing better than non-athletes, and they concluded that participation in sports is beneficial *per se* for the development of inhibitory control, resulting in greater SSRT performance in athletes. Furthermore, they also investigated different moderating variables and found that athletes' expertise level did not moderate SSRT performance. The authors argued that the type of sport could be relevant, because most studies in team sports found expertise effects (e.g., Verburgh et al., 2014; Hagyard et al., 2021), but studies in individual sport (e.g., Gutiérrez-Davila et al., 2013; van de Water et al., 2017) found no differences between different expertise levels. They concluded that advantages arise only in athletes who are acting in highly cognitive demanding environments with unpredictable and fast-changing situations such as team sports. Meng et al. (2019) supported this statement by showing that team sports athletes (volleyball) outperformed athletes from individual sports (badminton). They discussed the notion that the structure of the type of the sport may be relevant, and that more complex cognitive processes are involved in team sports. The subjects in the present study participated only in team sports (volleyball, ice hockey, basketball, handball, American football, soccer), so expertise effects could emerge. This is also in line with further studies showing greater performances in open-skill sports with higher demands on cognitive resources (Zhu et al., 2020). In addition, there are also some reviews suggesting superior benefits for open-skill sports in some aspects of cognitive functions (e.g., in executive functions) compared to closed skill sports (Gu et al., 2019; Heilmann et al., 2022).

Overall, the effects of our study are small. However, our participants did not cover the full range of expertise (1–16 points). Most athletes scored in the expertise range between 4 and 8 points, and no athlete could be classified as world class (above 12 points). This was somewhat surprising, because we recruited only athletes participating in the highest national league or in the national team. However, we also had players from the German junior national team who could not score high on some categories (e.g., on Category C: experience on the athlete's highest level) due to their young age. Nevertheless, this underlines two points: First, it is extremely difficult to recruit athletes on the highest level (world-class level); second, more studies are needed that classify elite athletes with higher resolution. At present, the definition of elite athletes is broad and varies between studies. To gain a clearer picture of expertise effects in response inhibition, but also in other cognitive domains, criteria of “eliteness” must be clear and transparent. Nonetheless, despite the low variance in expertise score, we still found a significant effect, thereby confirming a relationship between expertise score and response inhibition performance of the hands. It could be hypothesized that this effect will increase when the range of expertise scores is broader. This would be in line with Hagyard et al. (2021), because they also used Swann et al. (2015) classification frame, but with the full range of expertise scores. In their two studies, they recruited soccer, rugby, and basketball players on the highest level (superelite), elite level, and amateur level, and found shorter SSRTs for superelite athletes followed by elite athletes and amateur athletes. Therefore, findings indicate that SSRT performance improves with increased elite level, even with minor differences in expertise level such as in our study.

Furthermore, our results show a significant relationship between expertise scores and SSRTs only for hands. We found no significant results between expertise score and SSRTs for feet. These results are in line with a study by Heppe and Zentgraf (2019), which also showed significant results for hands but not for feet. The authors concluded that experts are not better *per se* in response inhibition, but rather benefit from participating in a type of sport that is typically hand-dominated (e.g., handball with catching or throwing) or foot-dominated (e.g., soccer with dribbling and shooting). The majority of our participants also engaged in hand-dominated sports such as volleyball (setting, hitting), basketball (throwing, dribbling, catching), or ice hockey (handling a stick). Hence, we are able to replicate the findings of Heppe and Zentgraf (2019), also showing a relationship between SSRTs for hands and expertise scores, but not for feet. On the other hand, because there was only one soccer player in our study, we cannot confirm that athletes who perform mainly with their feet (i.e., soccer actions such as shooting, dribbling, passing) will show similar results due to advantages for their feet. This is a limitation of our study. Further studies should evaluate more feet-dominated athletes (mainly soccer players) with different expertise level in order to address open questions in effector specificity.

Another open question is whether and how far inhibition performance is trainable. The literature (Anderson et al., 2001) shows that executive functions develop from early childhood into adulthood. In addition, previous studies indicate structural and functional changes in the brain due to training in (open-skill) exercise resulting in greater cognitive performance (Zhu et al., 2020). Also, a study in preadolescent soccer players suggests that inhibition performance seems to be trainable (Trecroci et al., 2022). This could suggest that inhibition performance is changing due to exposure and experience, impacting the relation between higher expertise levels and inhibition performance as found in our study. Hence, further research should address inhibition performance in different age groups and also carry out long-term intervention studies.

## 5. Conclusion

Results of our study suggest a differentiation in elite athletes regarding motor inhibition performance for hands. Also, another study (Heppe and Zentgraf, 2019) found differences with the hands, but not in the feet. In future studies the role of various sports that require greater use of the feet (e.g., soccer) or hands (e.g., handball) should be investigated. Nonetheless, the present study provides no information on whether expertise affects inhibition performance or vice versa. This should also be examined in further studies.

## Data availability statement

The raw data supporting the conclusions of this article will be made available by the authors, without undue reservation.

## Ethics statement

The studies involving human participants were reviewed and approved by Goethe University Frankfurt am Main, Department of Psychology and Sports Science, Ethics Committee. Written informed consent to participate in this study was provided by the participants' legal guardian/next of kin.

## Author contributions

M-TF and KZ designed the experiment. LR, BW, and M-TF performed the experiment. M-TF, LR, BW, and KZ analyzed and interpreted the data. M-TF wrote the initial draft of the manuscript. LR, BW, and KZ edited the manuscript. All authors contributed to drafting the work and revising it critically.
